# Chronic Dietary Supplementation of 4% Figs on the Modification of Oxidative Stress in Alzheimer's Disease Transgenic Mouse Model

**DOI:** 10.1155/2014/546357

**Published:** 2014-06-19

**Authors:** Selvaraju Subash, Musthafa Mohamed Essa, Abdullah Al-Asmi, Samir Al-Adawi, Ragini Vaishnav

**Affiliations:** ^1^Department of Food Science and Nutrition, College of Agriculture and Marine Sciences, P.O. Box 34, Sultan Qaboos University, Al-Khoud, 123 Muscat, Oman; ^2^Ageing and Dementia Research Group, Sultan Qaboos University, 123 Muscat, Oman; ^3^College of Medicine and Health Sciences, Sultan Qaboos University, 123 Muscat, Oman; ^4^Oman Assistant Pharmacy Institute, Directorate General of Education and Training, Ministry of Health, P.O. Box 1928, 114 Muttrah, Oman

## Abstract

We assessed the changes in the plasma A*β*, oxidative stress/antioxidants, and membrane bound enzymes in the cerebral cortex and hippocampus of Alzheimer's disease (AD) transgenic mice (Tg2576) after dietary supplementation of Omani figs fruits for 15 months along with spatial memory and learning test. AD Tg mice on control diet without figs showed significant impairment in spatial learning ability compared to the wild-type mice on same diet and figs fed Tg mice as well. Significant increase in oxidative stress and reduced antioxidant status were observed in AD Tg mice. 4% figs treated AD Tg mice significantly attenuated oxidative damage, as evident by decreased lipid peroxidation and protein carbonyls and restoration of antioxidant status. Altered activities of membrane bound enzymes (Na^+^ K^+^ ATPase and acetylcholinesterase (AChE)) in AD Tg mice brain regions and was restored by figs treatment. Further, figs supplementation might be able to decrease the plasma levels of A*β* (1–40, 1–42) significantly in Tg mice suggesting a putative delay in the formation of plaques, which might be due to the presence of high natural antioxidants in figs. But this study warrants further extensive investigation to find a novel lead for a therapeutic target for AD from figs.

## 1. Introduction

Alzheimer's disease (AD) is a progressive neurodegenerative disorder with complex multifactorial pathological changes in the brain. It is most prevalent form of dementia characterized by a progressive impairment of memory, cognitive functions, and behavior in the elderly [1]. It affects millions of people and has become major medical and social burden in developed and developing countries [2]. This disease has been reported to be the sixth leading cause of death. The neuropathology of AD is characterized initially by the deposition of senile plaques mainly composed of amyloid beta protein (A*β*) and neurofibrillary tangles containing hyperphosphorylated tau protein in the brain and later by the loss of neurons and their processes [3, 4]. Cognitive impairment appears to be most closely correlated in time with the loss of neurons and neuronal processes [5]. Accumulation of A*β* peptide might cause an increase in intracellular reactive oxygen species (ROS) and free radicals. The generation of ROS and relative oxidative damage is believed to be involved in the pathogenesis of AD. The ROS can induce functional and structural damage to cell membranes through lipid peroxidation and carbonyl modification of protein which may be involved in the pathogenesis of AD [6]. Other characteristic transforms that occur in AD are the increment of acetylcholinesterase (AChE) [7] around the amyloid plaques. The relationship between plaque/tangle deposition and the neuronal degeneration is not clearly understood. However, most of the AD cases occur sporadically, resulting from the influence of various nongenetic environmental factors. The mechanisms underlying AD appear to be diverse and so are the potential therapeutic methods for treating AD.

Currently, the interest in the role of dietary antioxidants in human health has been prompted in the area of neurodegenerative disease research. Fruits are good sources of bioactives, and there are a number of commercial polyphenol-rich beverages, which base their marketing strategies on antioxidant potency. Since the last decade, antioxidant has received a special attention as dietary supplements and several studies have shown inhibition of A*β* plaque formation* in vitro* and* in vivo* by natural compounds [8–13]. Curcumin and ginkgo biloba extract have been reported to have protective effect against the progression of AD pathology in AD murine models [14, 15].

The fig (*Ficus carica L*.) is a classical fruit tree associated with the beginnings of horticulture in the Mediterranean basin [16, 17]. Since ancient time, the Mediterranean region and the Middle East countries have been the most important cultivating centres of figs [18]. Compared with other common fruits and beverages, figs are an excellent source of minerals, vitamins, and dietary fiber; they are fat and cholesterol free and contain abundant amino acids [19–22]; it contains the highest concentrations of polyphenols [23]. The fig fruit is well known for its attractive taste and nutritive value due to its antioxidant properties, and it is consumed fresh or dried worldwide [21, 24–26]. The leaves are being used traditionally in the treatment of jaundice [27]. Figs are an excellent source of phenolic compounds, such as proanthocyanidins [23]. Actually, red wine and tea, two well-publicized sources of phenolic compounds, contain lower amounts of phenols than figs [28]. Figs have been reported to have excellent radical scavenging and antioxidant [21] activities. The effect of figs fruits on experimental AD is not yet well studied. To address this, we performed a set of experiments in a transgenic mouse model of AD supplemented with figs for 15 months with a focus on A*β* and oxidative stress.

## 2. Materials and Methods

### 2.1. Collection and Preparation

Fresh figs fruits were collected from Al-Jabal Al-Akhdar farms, Oman. The flesh was isolated manually, rinsed with water, dried for 18 h in a drying cabinet at 40°C, and stored at room temperature. The dried fruits will be crushed and extracted with acetone (1 : 1 ratio, weight to volume) under agitation at room temperature. After 48 h, the extract was then filtered and the filtrate will be evaporated to dryness in a drying cabinet at 40°C and stored. After that, the samples were ground into fine powder using a coffee grinder.

### 2.2. Diet Preparation for the Animals

The ground figs were sent to USA to prepare the diet for the mice. The diet was prepared by mixing the figs (4%) with regular diet as per National Institutes of Health, USA, protocol by Research Diet Inc., NJ, USA.

### 2.3. Animals and Treatment

Twelve transgenic female (APPsw/Tg 2576) and 6 wild control (nontransgenic) mice (Taconic form, NY, USA) were used. Animals were quarantined for 7 days after shipping and individually housed in plastic cages in an animal room, which was maintained at a temperature of 22 ± 2°C, a relative humidity of 50 ± 10%, and a 12 h light/dark automatic light cycle (light: 0800–2000 h). All these animals are free from pathogens and viruses. Experimental period commenced at the age of 4 months. The animals were divided into three groups: Group 1: wild-type (nontransgenic) control of the APPsw mice fed with regular diet, Group 2: AD transgenic mice also fed with regular diet, and Group 3: AD mice fed with 4% figs fruit diet. Influence of fig supplemented diet on cognitive behavior after 15 months was assessed by using the Morris water maze test (for spatial memory and learning ability). Following the behavioral assessments, oxidative stress, antioxidants, and membrane bound enzymes were investigated in experimental and control mice. All animal experiments in the present study were complied with the Animal Care and Use Committee of the Sultan Qaboos University, Oman (SQU/AEC/2010-11/3).

### 2.4. Blood and Tissue Sample Collection

The day after completion of the behavioral tests, blood samples were collected from all groups for plasma separation and all the samples were stored at −80°C until being used. Then the animals were decapitated with the head transferred onto the dry ice, followed by rapid dissection of the hippocampus and the cerebral cortex, homogenization in 9 volumes (1 : 9 w/v) of cold saline for preparation of a 10% cerebral homogenate in an ice bath, and centrifugation for supernatant collection. Whole brains were rapidly removed simultaneously and chilled in an ice-cold saline solution. The tissue samples were stored at −80°C until assay.

#### 2.4.1. Morris Water Maze Test

The water maze consisted of a metal pool (170 cm in diameter × 58 cm tall) filled with tap water (25°C, 40 cm deep) divided into four quadrants. In the centre of one quadrant was a removable escape platform below the water level covered with a nontoxic milk powder. The pool was divided into four quadrants (NE, NW, SE, and SW) by two imaginary lines crossing the centre of the pool. For each animal, the location of invisible platform was placed at the centre of one quadrant and remained there throughout training. The mice must memorize the platform location in relation to various environmental cues, and there was nothing directly indicative of the location of the escape platform in and outside of the pool. Therefore, the placement of the water tank and platform was the same in all acquisition trials. Each mouse was gently placed in the water facing the wall of the pool from one of the four starting points (N, E, S, or W) along the perimeter of the pool, and the animal was allowed to swim until it found and climbed onto the platform. During the training session, the mice subject was gently placed on the platform by an experienced investigator when it could not reach the platform in 60 s. In either case, the subject was left on the platform for 15 s and removed from the pool. The time for animals to climb onto the hidden platform was recorded as escape latency or acquisition time. In order to determine the capability of the animals to retrieve and retain information, the platform was removed 24 h later and the mice were released into the quadrant diagonally opposite to that which contained the platform. Time spent in the region that previously contained the platform was recorded as retention time. In each trial, the animal was quickly dried with a towel before being returned to the cage [29]. All tests were carried out at the end of the experimental period following 4% fig fruit dietary supplementation. ANY-maze software from Ugo Basile, Italy, was used.

#### 2.4.2. Determination of Plasma A*β* (1–40) and A*β* (1–42)

A*β*1–40 and A*β*1–42 plasma were measured by commercially available ELISA kits (Araclon Biotech Ltd., Zaragoza, Spain).

### 2.5. Biochemical Assays in the Brain

Oxidative stress markers such as malondialdehyde (MDA) [30] and total protein carbonyl content [31–33] were assayed in hippocampus and the cerebral cortex. The activities of enzymatic antioxidants, superoxide dismutase (SOD) [34], CAT [35], glutathione peroxidase (GPx) [36, 37], glutathione reductase (GR) activity [38], and the levels of reduced glutathione (GSH) [39] were also analyzed in hippocampus and the cerebral cortex. Furthermore, the activities of membrane bound enzymes such as acetylcholinesterase (AChE) activity [40] and Na^+^ K^+^ ATPase [41, 42] were also assayed in hippocampus and the cerebral cortex. Protein estimation was conducted according to Lowry et al. [43].

### 2.6. Statistical Analysis

The statistical analysis was performed using SPSS software version 16.0. The results were expressed as mean ± SEM. All data were statistically analyzed by one-way analysis of variance (ANOVA), followed by Dunnett's *t*-test. A significant difference was determined when *P* < 0.05.

## 3. Results

### 3.1. A 4% Fig Rich Diet Improved Spatial Memory in AD Tg Mice

**Figure 1:**
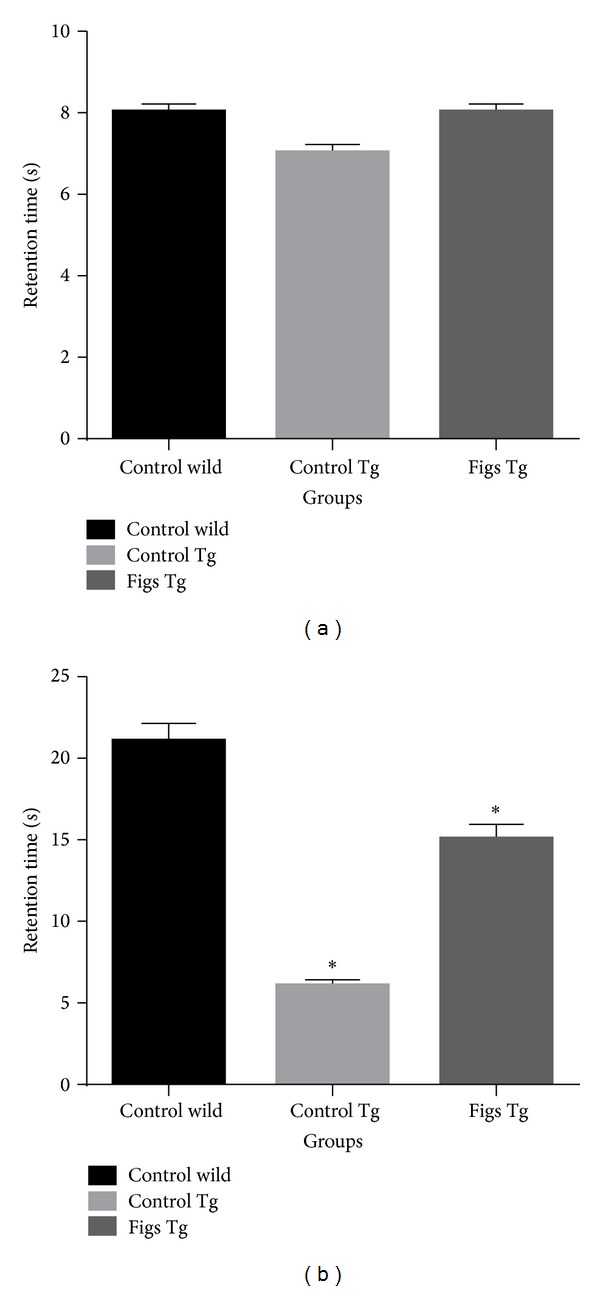
Supplementation with 4% figs ameliorated the decline in spatial memory and learning ability of Tg mice. (a) Retention time in Morris water maze test at the age of 4 months. (b) Retention time in Morris water maze test after treatment of figs diet for 15 months. Data are presented as mean ± SD and *n* = 6/group. *P < 0.05 compared to wild-type mice.

The cognitive ability of the Tg mice was assessed by the Morris water maze test. Wild-type control mice after 15 months were given the task of learning how to find the hidden platform in the Morris water maze, and their performance was found to improve in an experience-dependent manner. In contrast, the Tg mice after 15 months showed a significantly delayed latency to finding the hidden platform compared with the wild control mice (Figures 1(a) and 1(b)). Figs supplementation to Tg mice for 15 months significantly improved the escape latency to find the platform than Tg mice on control diet (Figures 1(a) and 1(b)), which indicates that figs might be able to improve spatial memory in Tg mice.

### 3.2. Effect of 4% Figs on A*β* (1–40) and A*β* (1–42) Content in Plasma

Plasma levels of both A*β*1–40 and A*β*1–42 were significantly higher in Tg mice on normal diet than wild-type mice on the same diet and figs supplementation ameliorated these levels significantly (Table 1) than Tg mice on normal diet.

### 3.3. Effect of 4% Figs on LPO and Protein Carbonyls in AD Transgenic Mice

APPsw (Tg2576) AD mice showed significant increase in LPO levels in both brain regions studied (cortex and hippocampus) compared to wild type (Table 2). However, 4% figs dietary supplemented AD mice for 15 months attenuated the increase in LPO comparable to wild control values. Table 1 depicts significantly elevated levels of protein carbonyls in disease control APPsw (Tg2576) mice compared to wild type (cortex and hippocampus) and 4% figs dietary supplementation significantly brings down protein carbonyl levels in AD mice.

### 3.4. Effect of 4% Figs on the Antioxidant Enzymes in APPsw (Tg2576) AD Transgenic Mice

Significantly decreased activities of SOD, GPX, GR, and CAT in cerebral cortex and hippocampus were found in AD mice when compared to wild mice (Tables 3 and 4). However, the entire antioxidant enzyme activities were significantly enhanced by 4% figs dietary supplementation in cerebral cortex and hippocampus of APPsw (Tg2576) AD mice.

GSH activity in brain regions of disease control APPsw (Tg2576) mice was significantly decreased in cortex and hippocampus compared to wild-type mice (Table 3). However, 4% figs dietary supplemented mice restored GSH activity to near normal levels in cortex and hippocampus.

### 3.5. Effect of 4% Figs on Membrane Bound Enzymes in AD Transgenic Mice

AChE activity significantly increased in the cortex and hippocampus of control APPsw (Tg2576) mice. Dietary supplementation of 4% figs for 15 months attenuated AChE activity in the cerebral cortex and hippocampus of AD mice. Disease control APPsw (Tg2576) mice showed significant inhibition in Na^+^ K^+^ ATPase activity in the cortex and hippocampus and figs dietary supplementation could be able to offer a significant improvement in the activity of membrane bound enzymes (Table 5).

## 4. Discussion

To our knowledge, this study is the first to investigate the ability of figs to attenuate oxidative stress in AD transgenic mice. Our current results clearly demonstrated that dietary supplementation of figs could significantly improve the learning and memory deficits in AD transgenic mice. Figs diet fed mice spend more time in the target quadrant and made more annulus crossings than the animals fed with the control diet during the probe test [44].

Previous studies proposed a model for neurodegeneration in AD brains based on free radicals/oxidative stress associated with A*β* (1–40 and 1–42) [45, 46]. The increased levels of plasma A*β* in AD were also previously documented [47, 48]. It has been observed that AD transgenic mice could secrete more A*β*1–42 and A*β*1–40 than their wild control littermates throughout their life [49, 50], which coincides with our results. But the effect of figs diet on reducing the plasma A*β* (1–40 and 1–42) levels in Tg mice shows that figs may offer beneficial effect before A*β* plaque formation.

ROS can damage essential cellular constituents such as lipids and proteins, which can be measured by identification of their by-products MDA and protein carbonyl, respectively [51]. We observed increase of MDA and production and protein carbonylation in cerebral cortex and hippocampus of AD Tg mice, indicating that oxidative stress occurs as a consequence of AD, thereby contributing to brain damage. Dietary supplementation of figs notably inhibited the accumulation of MDA and protein carbonyl levels in cortex and hippocampus of Tg mice, which is an oxidized by-product of lipid peroxidation. This context was supported by the previous studies that the figs and figs leaves could reduce MDA level, an index of lipid peroxidation [27] on carbon tetrachloride induced rats [52].

GSH offers primary defense in neurons against oxidative stress and maintains cellular redox homeostasis [53]. In our experiment we observed a significant decrease in the GSH levels in the brain of AD Tg mice compared to wild controls. It is known that GSH depletion is the first indicator of oxidative stress during neurodegenerative diseases [54]. Figs supplementation to AD Tg mice was able to reverse the decrease in GSH levels. Aziz [55] has reported that figs could be able to reverse the GSH levels in lead acetate and carbon tetrachloride induced hepatotoxicity [52] in rats, suggesting the efficacy of figs in preventing the oxidative damage and associated changes.

SOD is responsible for catalyzing the conversion of superoxide anions into hydrogen peroxide [56, 57] which is further decomposed to water and oxygen by CAT [58]. The activities of SOD and CAT were found to be significantly diminished in cortex and hippocampus of AD Tg mice. Figs supplementation in diet to AD Tg mice prevented decrease in the activities of SOD and CAT. Studies have shown that figs could directly inhibit the superoxide anion formation which could enable the restoring of SOD and CAT and our result suggests that the neuroprotective effects of the figs might be due to their antioxidant activity [52, 55, 59].

GPx and GR represent a crucial defensive system to protect cells against ROS [60]. The activity of GPx and GR was significantly decreased in brain regions. Isharat et al. [61] have reported significant decrease in these enzymes in experimental dementia. On other hand, Fan et al. [62] have reported the decreased GPx and GR activity as result of oxidative stress in scopolamine induced amnesia in the hippocampus and cerebral cortex. Figs supplementation significantly attenuated the elevated levels of GPx and GR in brain regions. Figs have shown to be successful in increasing the activity of GPx and GR enzyme in rats with oxidative damage induced by methanol [59].

AChE is an acetylcholine hydrolyzing enzyme that is responsible for the termination of cholinergic response [63]. The AChE activity was found to be markedly elevated in Tg mice brain regions. This observation coincides with previous reports whereby I.C.V. administration of streptozotocin at subdiabetogenic dose has been shown to induce memory deficits along with increase in oxidative stress and AChE activity [64, 65]. AChE activity was significantly increased in hippocampus in L-methionine induced model of vascular dementia [66] and the activity of AChE depends largely on the membrane characteristics, since the enzyme is membrane bound. Barbosa et al. [67] suggested that amyloid beta peptides induce Ca^2+^ influx that leads to increased activity of AChE which is attributed to Ca^2+^ mediated oxidative stress. In our study, dietary supplementation of figs could be able to inhibit AChE activity which was supported by the previous study suggesting that leaf extracts of fig could offer AChE inhibitory activity and antioxidant effects [68]. But the mechanism of anticholinesterase activity reduction by figs appears to be complicated and needs further extensive investigation.

Modification of Na^+^ K^+^ ATPase activity may induce neuronal death with features of both apoptosis and necrosis [69]. In the current study, the activity of Na^+^ K^+^ ATPase was found to be decreased in AD Tg mice, which is in line with other studies reporting decrease in the enzyme activity during aging [70, 71]. Na^+^ K^+^ ATPase is known to be highly susceptible to changes in the membrane lipids, which may be further attributed to the progressive increase in the lipid peroxidation [72, 73]. ROS overproduction inhibits the activity of ATPase via thiol- and lipid-dependent mechanisms [74]. It has been demonstrated that the reduced activity of Na^+^ K^+^ ATPase caused by oxidative stress cannot drive the ion pumps to maintain depolarization of neurons and thus may become lethal to neurons [74]. The mechanism of the action of enhancing Na^+^ K^+^ ATPase effects of figs is uncertain, as its multiple active compounds such as anthocyanins, coumarins, caffeoylquinic acids, ferulic acid, quercetin, fumaric acids, alkaloids, and flavonoids have multifunctional action, making it complex in pharmacological action. Recently we have reported that the date fruits could offer protection to oxidative damage in AD mice [75]. Further inhibition in lipid peroxidation and enhancement of antioxidant enzymes in various disease conditions by figs have already been reported also support our findings [21, 22, 76].

## 5. Conclusion

In conclusion, figs could improve memory related behavioral deficits, reducing the A*β* and oxidative damage and enhancing the antioxidant system in AD transgenic mice. Protection from A*β* mediated oxidative damage in brain could be potentially considered as a promising strategy for therapeutic intervention in AD. Our results allow us to conclude that figs diet seems to be an effective modifying therapeutic strategy for AD. Further extensive experiments must be done in order to elucidate the molecular mechanisms through which figs diet may mediate its beneficial effect on AD like neurodegenerative disease condition.

## Figures and Tables

**Table 1:** Figs diet effect on plasma A*β* (1–40, 1–42) levels in Tg mice.

Groups	Plasma A*β* levels (pg/mL)	
	A*β*1–40	A*β*1–42
Control wild	82.01 ± 6.25^a^	98.02 ± 7.46^a^
Control Tg	1354.68 ± 103.69^b^	282.14 ± 21.60^b^
4% Figs Tg	840.28 ± 64.17^c^	164.05 ± 12.53^c^

Data are presented as mean ± SD, and *n* = 6/group.

Values not sharing common superscripts (a, b, and c) differ significantly at *P* < 0.05 (DMRT).

**Table 2:** Effect of figs on lipid peroxidation and protein carbonyls content in brain of Tg mice.

Groups	MDA levels (nmol/mg protein)		Protein carbonyl (nmol/mg protein)	
	Cortex	Hippocampus	Cortex	Hippocampus
Control wild	1.60 ± 0.12^a^	0.90 ± 0.07^a^	30.01 ± 0.06^a^	51.01 ± 0.06^a^
Control Tg	3.60 ± 0.28^b^	2.10 ± 0.16^b^	81.26 ± 0.05^b^	132.14 ± 0.05^b^
Figs Tg	2.70 ± 0.21^c^	1.40 ± 0.11^ c^	49.86 ± 4.61^ c^	101.23 ± 4.61^c^

Data are presented as mean ± SD, and *n* = 6/group.

Values not sharing common superscripts (a, b, and c) differ significantly at *P* < 0.05 (DMRT).

**Table 3:** Effect of figs on superoxide dismutase and catalase activity in cortex and hippocampus of Tg mice.

Groups	SOD (U/mg protein)		Catalase (U/mg protein)	
	Cortex	Hippocampus	Cortex	Hippocampus
Control wild	196.03 ± 14.93^a^	215.04 ± 16.37^a^	3.80 ± 0.29^a^	4.30 ± 0.33^a^
Control Tg	114.06 ± 8.73^b^	128.06 ± 9.80^b^	1.70 ± 0.13^b^	2.20 ± 0.17^b^
Figs Tg	165.06 ± 12.61^c^	179.06 ± 13.67^ c^	2.90 ± 0.22^ c^	3.40 ± 0.26^c^

Data are presented as mean ± SD, and *n* = 6/group.

Values not sharing common superscripts (a, b, and c) differ significantly at *P* < 0.05 (DMRT).

**Table 4:** Effect of figs dietary supplementation on glutathione dependent antioxidant enzymes in the brain of Tg mice.

Groups	Glutathione peroxidase (nmol NADPH oxidized/min/mg protein)		GSH (mg/g protein)		Glutathione reductase (nmol NADPH oxidized/min/mg protein)	
	Cortex	Hippocampus	Cortex	Hippocampus	Cortex	Hippocampus
Control wild	12.00 ± 0.91^a^	24.00 ± 1.83^a^	4.20 ± 0.32^a^	5.80 ± 0.44^a^	11.00 ± 0.84^a^	11.70 ± 0.89^a^
Control Tg	5.00 ± 0.38^b^	11.01 ± 0.84^b^	2.10 ± 0.16^b^	3.20 ± 0.25^b^	4.00 ± 0.31^b^	4.60 ± 0.35^b^
Figs Tg	9.10 ± 0.70^c^	17.01 ± 1.30^ c^	3.20 ± 0.24^ c^	4.70 ± 0.36^c^	8.20 ± 0.63^c^	9.00 ± 0.69^c^

Data are presented as mean ± SD, *n* = 6/group.

Values not sharing a common superscripts (a, b and c) differ significantly at *P* < 0.05 (DMRT).

**Table 5:** Influence of figs dietary supplementation on AChE and Na^+^ K^+^ ATPase activity in cortex and hippocampus of Tg mice.

Groups	AChE (U/mg Protein)		Na^+^ K^+^ ATPase (% control)	
	Cortex	Hippocampus	Cortex	Hippocampus
Control wild	2.40 ± 0.06^a^	2.80 ± 0.06^a^	98.02 ± 7.46^a^	97.02 ± 7.39^a^
Control Tg	4.40 ± 0.05^b^	4.80 ± 0.05^b^	36.01 ± 2.74^b^	32.01 ± 2.44^b^
Figs Tg	3.20 ± 0.06^c^	3.60 ± 0.06^ c^	70.01 ± 5.33^ c^	69.01 ± 5.25^c^

Data are presented as mean ± SD, and *n* = 6/group.

Values not sharing common superscripts (a, b, and c) differ significantly at *P* < 0.05 (DMRT).
